# Construction of an Immune-Related Six-lncRNA Signature to Predict the Outcomes, Immune Cell Infiltration, and Immunotherapy Response in Patients With Hepatocellular Carcinoma

**DOI:** 10.3389/fonc.2021.661758

**Published:** 2021-07-02

**Authors:** Pengcheng Zhou, Yuhua Lu, Yewei Zhang, Lei Wang

**Affiliations:** ^1^ Southeast University Medical School, Nanjing, China; ^2^ Department of Hepatobiliary and Pancreatic Surgery, Affiliated Hospital of Nantong University, Nantong, China; ^3^ Department of Hepatobiliary and Pancreatic Surgery, Southeast University Zhongda Hospital, Nanjing, China; ^4^ Department of Hepatobiliary and Pancreatic Surgery, The Second Affiliated Hospital of Nanjing Medical University, Nanjing, China

**Keywords:** Hepatocellular carcinoma, lncRNA, immune, TCGA, riskScore, immunotherapy

## Abstract

**Background:**

Hepatocellular carcinoma (HCC) is one of the world’s most lethal malignant tumors with a poor prognosis. Growing evidence has been demonstrating that immune-related long non-coding RNAs (lncRNAs) are relevant to the tumor microenvironment (TME) and can help assess the effects of immunotherapy and evaluate one’s prognosis. This study aims to identify an immune-related lncRNA signature for the prospective assessment of the immunotherapy and prognosis in HCC.

**Method:**

We downloaded HCC RNA-seq data and clinical information from The Cancer Genome Atlas (TCGA) project database. We first used ESTIMATE to evaluate the TME. Then, we conducted a cox regression analysis to construct a prognostic signature and the riskScore. We then applied the univariate Cox regression, multivariate Cox regression, principal components analysis (PCA), receiver operating characteristic (ROC) curve, and stratification analyses to confirm our previous assessments. Afterward, we employed a gene set enrichment analysis (GSEA) to explore the biological processes and pathways. Besides, we used CIBERSORT to estimate the abundance of tumor-infiltrating immune cells (TIICs). Furthermore, we investigated the relationship between the immune-related lncRNA signature and immune checkpoint genes. Finally, we used the quantitative real-time polymerase chain reaction (qRT-PCR) assays to demonstrate the expression of the six lncRNAs.

**Results:**

We identified six immune-related lncRNAs — MSC-AS1, AC145207.5, SNHG3, AL365203.2, AL031985.3, NRAV — which show the ability to stratify patients into high-risk and low-risk groups with significantly different survival rates. The univariate Cox regression, multivariate Cox regression, ROC, and stratification analyses confirmed that the immune-related six-lncRNA signature was a novel independent prognostic factor in HCC patients. The high-risk group and low-risk group illustrated contrasting distributions in PCA. The GSEA suggested that the immune-related six-lncRNA signature was involved in the immune-related biological processes and pathways. Besides, the immune-related six-lncRNA signature was associated with the infiltration of immune cells. Furthermore, it was linked with the expression of critical immune genes and could predict immunotherapy’s clinical response. Finally, the qRT-PCR demonstrated that the six lncRNAs were significantly differentially expressed in HCC cell lines and normal hepatic cell lines.

**Conclusion:**

In summary, we identified an immune-related six-lncRNA signature that can predict the outcomes, immune cell infiltration, and immunotherapy response in patients with hepatocellular carcinoma.

## Background

Hepatocellular carcinoma (HCC) is one of the most common human malignancies and the fourth most common cause of cancer mortality after lung, colorectal, and stomach cancers, according to the World Health Organization (WHO). HCC exerts a heavy disease burden, with more than 800,000 newly diagnosed cases and nearly 700,000 deaths each year (1). Due to its aggressive and insidious growth nature, most patients are diagnosed at advanced stages, at which point therapeutic options are limited and ineffective. Although we made many advances in the diagnostic and therapeutic strategies of HCC, the outcomes for HCC patients remain unfavorable. Indeed, the median survival rate of advanced HCC patients is about nine months and the 5-year overall survival rate is only 10% (2). Considering the high mortality rate, we must further study the clinical diagnosis methods of HCC, explore new risk factors and molecular markers, and develop new therapeutic targets, which could improve the clinical prognosis of patients suffering from HCC.

In recent years, the continuous progress of gene and molecular biology technology has revealed that the TME plays a vital role in tumor epigenetics, tumor differentiation, immune escape, and infiltration metastasis (3). Besides, emerging evidence has confirmed that disorders of the immune response in the TME play a pivotal role in cancer development and progression (4). Meanwhile, the expression and dysregulation of immune-related genes are implicated in the regulation of the immune system. The host immune response and immune cells, both crucial factors of the TME, are consistently involved throughout the tumor development (3). HCC shows a high degree of malignancy and its poor overall survival outcomes are due to the collapse of the patient’s immune surveillance. Therefore, a better understanding of immune-related factors in the TME and their effects on cancer cells will help discover novel prognostic elements of HCC.

Long non-coding RNAs (lncRNAs) are RNA molecules with transcripts longer than 200 nucleotides, which are frequently dysregulated in various cancers. Abundant studies have reached a consensus on lncRNAs’ involvement in cancer cells’ proliferation, migration, invasion, apoptosis, angiogenesis, and drug resistance. The dysregulation of numerous lncRNAs has been reported in different cancers, including breast and lung cancers, HCC, and many more. For example, HOTAIR, a 2,158 bp lncRNA, is more highly expressed in HCC tissues than in non-tumor tissues; promotes cell proliferation, autophagy, and invasion; and reduces the response of hepatoma cells to the apoptosis stimulator TNF-α and chemotherapeutic drugs (5). In addition to affecting cancer cells themselves, lncRNAs also influence the TME (6). In the TME, lncRNAs contribute to mediating and controlling several immune and cancer cell interactions, as well as the important mechanisms of the immune response (7). Additionally, lncRNAs significantly affect the tumor’s immune process and the infiltration of TIICs. Thus, we eagerly anticipate the discovery of some promising prognostic immune-related lncRNA markers and the investigation of the underlying molecular mechanisms.

In this study, we identified the expression of immune-related lncRNAs in 374 HCC patients from TCGA’s project database. Using the ESTIMATE of the TME, survival analysis, Cox regression model, CIBERSORT of TIICs, and other methods, we identified a biologically relevant six-lncRNA signature capable of predicting the prognosis of patients suffering from HCC. Finally, using a quantitative real-time polymerase chain reaction (qRT-PCR), we verified that these six lncRNAs’ expressions were significantly different between HCC cell lines and liver cell lines. We aimed to take advantage of the lncRNA expression profiles to explore immune-related lncRNAs, which may have potential clinical significance in patient management and shed light on the tumorigenesis of HCC.

## Methods

### Acquisition of HCC Expression Data

The human HCC transcriptome RNA-sequencing data were downloaded from TCGA (https://portal.gdc.cancer.gov/). We also downloaded the corresponding clinical information, such as patients’ genders, ages, and survival information from TCGA. The data was updated on June 2, 2020. The RNA-sequencing data were combined into an mRNA matrix file using the programming language Perl (http://www.perl.org/). Then, we converted genes’ Ensembl IDs into gene names. The RNA-sequencing data was combined into a mRNA matrix file by a merge script in the Perl programming language (http://www.perl.org/). Then the Ensembl IDs of genes were converted into gene names and lncRNAs were distinguished from mRNAs according to the biotype with the Ensembl database (http://asia.ensembl.org/index.html) by script in the Perl programming language.

### Evaluation of Tumor Microenvironment Infiltration Patterns

For each HCC dataset, we used single-sample gene-set enrichment analysis (ssGSEA) score to quantify the enrichment levels of 29 immune gene sets (8). HCC patients were hierarchically into high immune cell infiltration group and low immune cell infiltration group. We applied the ESTIMATE method to evaluate the presence of stromal cells and immune cells in the TME by calculating specific gene expression data (9). We also utilized the ESTIMATE algorithm, *via* the R software (https://cran.r-project.org/mirrors.html), to evaluate the tumor microenvironment of each HCC sample. These samples were then classified into high immune cell infiltration and low immune cell infiltration groups, and we calculated the EstimateScore, ImmuneScore, StromalScore, and TumorPurity.

### Analysis of Tumor Infiltrating Immune Cells

We applied the CIBERSORT method with absolute mode to estimate the abundance of TIICs based on the gene expression data (10). The CIBERSORT R package was used to calculate the proportion of 22 immune cell types in each sample.

### Acquisition of Immune-Related lncRNAs

We acquired the immune-related genes from the Molecular Signatures Database v 7.1 (Immune response M19817, immune system process M13664, http://www.broadinstitute.org/gsea/msigdb/index.jsp). Then, the immune‐related lncRNAs was identified by a Pearson correlation analysis between immune-related genes and lncRNA expression level in samples with correlation coefficient >0.5 and p < 0.001.

### Acquisition of Survival-Related lncRNAs

We combined the immune-related lncRNA expression with survival data (excluding samples with overall survival of ≤ 30 days). The survival-related lncRNAs were extracted through a univariate cox regression analysis, using the “survival” R package, with a significant prognostic value P < 0.0001 as the criteria.

### Construction of the Immune-Related lncRNA Signature Model

We conducted a multivariate Cox regression analysis to construct a prognostic signature, and calculated the risk score. The risk score for each patient was as follows: risk score = (lncRNA1 expression × coefficient lncRNA1) + (lncRNA2 expression × coefficient lncRNA2) + …+ (lncRNAn expression × coefficient lncRNAn). The risk score model was used as a measure of prognostic risk for each hepatic cancer patient. The median risk score served as a cutoff value to classify the patients into a high- and a low-risk group for the following study.

### Validation of the Immune-Related lncRNA Model

The R package “survival” and “survminer” were used to plot Kaplan–Meier survival curves to compare the survival difference for both groups with log-rank test. We utilized the receiver operating characteristic curve (ROC) to examine the performance of the survival-related lncRNAs. The R package “survivalROC” was used to investigate the prognostic value of the immune-related lncRNA signature. The univariate and multivariate Cox regression analysis was used to evaluate the prognostic relationship between risk score and age, gender, grade, clinical stage and TMN stage and the R package “ggpubr” was used to investigate the relationships between immune−related lncRNAs and clinical parameters with wilcox test.

### Principal Components Analysis

The principal components analysis (PCA) was carried out to demonstrate the expression patterns of immune-related lncRNAs in low-risk and high-risk groups.

### Role of Immune-Related lncRNA Signature on the Immunologic Features

We used the gene set enrichment analysis (GSEA) to detect the potential functional phenotypes or pathways in which immune-related lncRNAs may be involved. In the current study, we analyzed the gene sets of GO (gene ontology), KEGG (Kyoto Encyclopedia of Genes and Genomes), all immunologic signatures gene, all oncogenic signatures gene, immune response, and immune system process, using GSEA 4.0.3.

### Correlation Analysis of Immune Cell Infiltration

To investigate the immune function of lncRNAs in immune response, we performed a correlation analysis between lncRNAs expression and the landscape of infiltrating immune cells in HCC samples with CIBERSORT, xCell and ssGSEA. Firstly, we associated the immune-related lncRNA signature with 22 TIICs to figure out whether or not this immune-related lncRNA signature may play a crucial role in immune infiltration in HCC with CIBERSORT using absolute mode. Then, we used the “complexpheatmap” R package to generate the 22 TIICs’ heatmap. We also performed a spearman correlation analysis to evaluate the abundance of TIICs and their risk score. Secondly, we used xCell (11) to investigate the cellular heterogeneity landscape of HCC patients divided by lncRNA signature. Then, we used the “heatmap” R package to generate the 64 cells’ heatmap. We also performed a spearman correlation analysis to evaluate the abundance of 64 cells and the risk score. Thirdly, we evaluate 24 immune cells of each lncRNA with ssGSEA (12). The “GSVA” R package and spearman method was used to generate the figure. Samples with a output value P < 0.05 are considered significant.

### Cell Culture and Quantitative Real−Time PCR Detection of lncRNA Expressions in Cell Lines

Human HCC cell lines (Hep3B, HuH7, Li7) were purchased from the Type Culture Collection of the Chinese Academy of Sciences (Shanghai, China), HCCLM3 was purchased from Procell (Wuhan, China) and one normal human hepatic cell line (LO2) was purchased from Immocell (Xiamen, China). Cells were cultured in Dulbecco’s modified Eagle’s medium (DMEM, Hylcone) containing 10% FBS (Gibco). The cells were maintained in a humidified incubator at 37°C with 5% CO2. Total RNA was isolated from cells using the Trizol Reagent (Invitrogen) according to the manufacturer’s instructions. Each RNA was reverse-transcribed into cDNA with the High Capacity cDNA Synthesis kit (Applied Biosystems, Foster City, CA, USA). QRT-PCR was determined using the SYBR Premix Ex Taq kit (Takara Biotechnology Co., Ltd.) on a 7500 Fast Real−time PCR system (Thermo Fisher Scientific Inc., UK). PCR primer sequences were designed and synthesized by Sangon Biotech (Shanghai) Co., Ltd. The primer sequences for qRT-PCR are shown in **Table 1**. GAPDH was used as endogenous control. The relative expression levels are calculated as a fold change using 2-ΔΔCt method and the difference between two samples was calculated with T test.

**Table 1:** The primer sequences of six immune-related lncRNAs.

MSC-AS1	F primer (5′-3′)	AAGCAACAACTGTCTGGCCT
	R primer (5′-3′)	TGATGCCAGCAAATTGGTGC
AC145207.5	F primer (5′-3′)	GACTGGCCAAGCATTTGGTG
	R primer (5′-3′)	TCTGGCCTACCTTAGGCTACAT
SNHG3	F primer (5′-3′)	GGACCGTAAGTCTGGGTTGA
	R primer (5′-3′)	TACAACCTCCCGTTGCTACC
AL365203.2	F primer (5′-3′)	CTCGATGGGAAGACAGTGGT
	R primer (5′-3′)	TGGGATTTCTCCTTCACCTG
AL031985.3	F primer (5′-3′)	TGTGGTCCCTGTCACACCTA
	R primer (5′-3′)	AGAAGCCAAGGATTCCCCTA
NRAV	F primer (5′-3′)	GTTCTTGGCCATCGTGATCT
	R primer (5′-3′)	GGATGAGGTGAGGAGAGCTG
GAPDH	F primer (5′-3′)	ACAACTTTGGTATCGTGGAAGG
	R primer (5′-3′)	GCCATCACGCCACAGTTTC

F primer, forward primer; R primer, reverse primer.

### Statistical Analysis

All analyses were carried out using the R version 3.6.2 and the corresponding packages. P values < 0.05 were considered significant (*P < 0.05, **P < 0.01, ***P<0.001).

## Results

### The Immune Landscape of the TME in HCC

**Figure 1:**
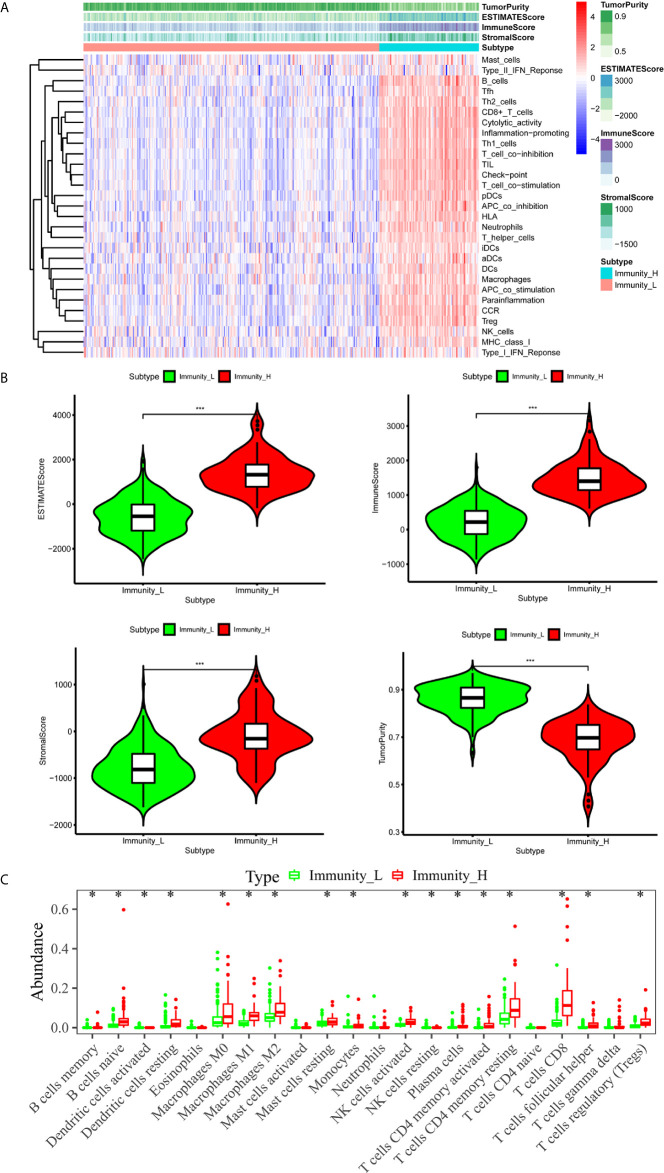
The immune landscape of the TME in HCC. **(A)** The heatmap showed that the immunity high group had lower Tumor Purity but higher ESTIMATE Score, Immune Score and Stromal Score. The TME of the two groups was significantly different. **(B)** The box chart showed that there was a significant positive correlation between immunity high group and ESTIMATE Score, Immune Score and Stromal Score, respectively, while there was a positive correlation between immunity low group and Tumor Purity. **(C)** Absolute CIBERSORT algorithm revealed that the abundance levels of immune cells differ in the immunity high group and immunity low group. *p < 0.05, **p < 0.01, ***p < 0.001.

We downloaded both transcriptome and clinical data from the TCGA database. The transcriptome data contained 50 normal samples and 374 tumor samples and the clinical data contained 377 HCC patients. We converted the Ensembl IDs of genes into gene names. The 29 immune gene sets represented diverse immune cell types, immune-related pathways, and immune-related functions (**Supplementary Table 1**). According to the results of the hierarchical clustering algorithm, HCC samples were divided into two groups, according to immune infiltration, including the high immune cell infiltration (n=94) and low immune cell infiltration (n=280) groups. Subsequently, we scored the TME of each sample and compared the TME’s characteristics, including the EstimateScore, ImmuneScore, StromalScore, and TumorPurity in the groups showing high and low levels of immunity. The heatmap showed that the group showing high levels of immunity had lower Tumor Purity but higher ESTIMATE, Immune, and Stromal Scores (**Figure 1A**). The TME of the two groups showed significant differences. The box chart (**Figure 1B**) also showed a significant positive correlation between the group showing high levels of immunity and the ESTIMATE, Immune, and Stromal Scores, respectively, while showing a positive correlation between the group showing low levels of immunity and Tumor Purity. To further explore the relationship between the high and low immunity levels of the TME and the infiltration of immune cells, we used the CIBERSORT algorithm to measure the relative proportions of immune cells in groups showing high and low levels of immunity. This research revealed that the abundance levels of B cells memory, B cells naive, Dendritic cell resting, Macrophages M0, Macrophages M1, Macrophages M2, Mast cells resting, Monocytes, NK cells activated, Plasma cells,T cells CD4 memory activated, T cells CD4 memory resting, T cells CD8, T cells follicular helper, and T cells regulatory (Tregs) were significantly higher in the group showing high levels of immunity than in the one showing low levels of immunity. Conversely, NK cells resting was significantly lower in the immunity high group (**Figure 1C**).

### Immune-Related lncRNAs in HCC

We divided the obtained genes into lncRNAs and mRNAs. We obtained a total of 331 immune-related genes from the Molecular Signatures Database v 7.1 (Immune response M19817, immune system process M13664), and identified 236 immune-related lncRNAs using the correlation analysis.

### The Relationships Between Immune-Related lncRNAs and Prognosis

**Figure 2:**
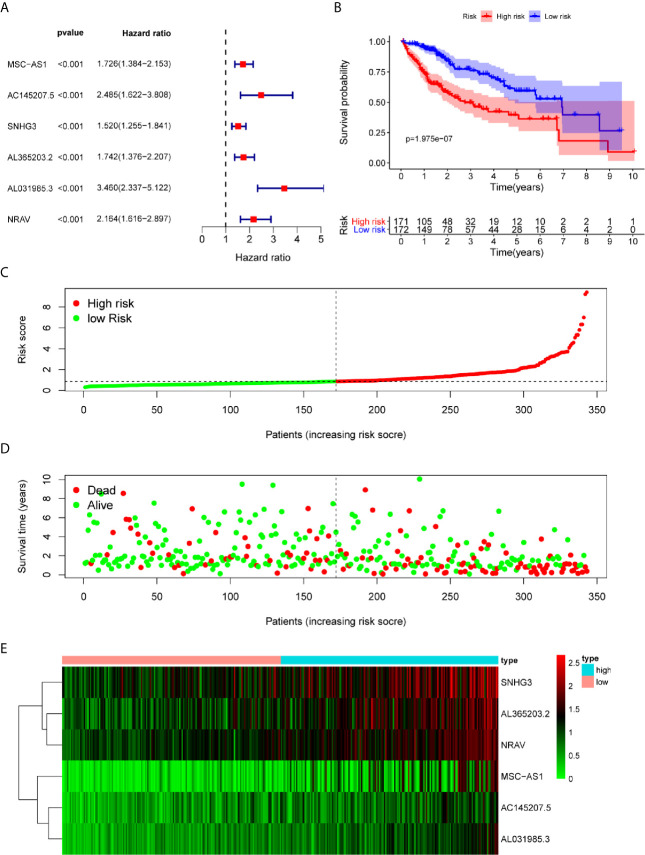
Construction and assessment of immune-related lncRNA prognostic signature for HCC. **(A)** The HR and p-value from the univariable Cox regression of selected immune-related lncRNAs (MSC-AS1, AC145207.5, SNHG3, AL365203.2, AL031985.3, NRAV) according to the criterion of p < 0.0001. **(B)** Kaplan-Meier survival curves showed that the overall survival of the high-risk groups was significantly lower than that of the low-risk groups. **(C)** HCC samples were divided into high-risk groups and low-risk groups according to the median value of risk score. **(D)** A higher mortality was observed in high-risk groups than in low-risk groups. **(E)** The heatmap showed that with the increase of risk score, the expression levels of lncRNAs were elevated.

A total of 343 HCC patients were included in the prognosis analysis, patients without transcriptome data or overall survival < 30 days were excluded. Using the univariate Cox regression, we identified six immune-related lncRNAs (MSC-AS1, AC145207.5, SNHG3, AL365203.2, AL031985.3, NRAV), which were associated with a prognosis according to the criterion of p < 0.0001. The forest map illustrated the relationships between these lncRNAs and the prognosis (**Figure 2A**).

### Validation of the Immune-Related lncRNAs Signature in HCC Survival

We calculated each patient’s risk score and divided the samples into high-risk (n=171)and low-risk (n=172) groups, according to the median value of the risk score (**Figure 2C**). We observed a higher mortality rate in high-risk groups than in low-risk groups (**Figure 2D**). The heatmap also showed that with the increase of risk score, the expression levels of lncRNAs were elevated (**Figure 2E**). The Kaplan-Meier survival curves showed that the overall survival of the high-risk group was significantly lower than that of the low-risk group, indicating the effectiveness of the immune-related lncRNAs signature (p = 1.975e-07) (**Figure 2B**). Collectively, these studies identify six immune-related lncRNAs as a prognostic signature for HCC.

### Evaluation of Immune-Related lncRNAs as Independent Prognostic Factors in HCC

**Figure 3:**
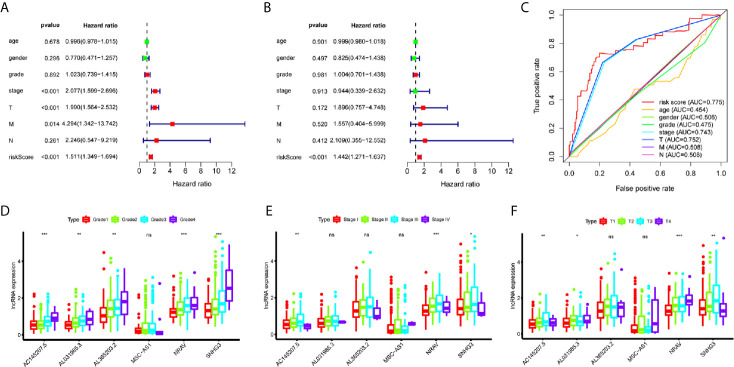
Evaluation of immune-related lncRNAs as independent prognostic factors in HCC. **(A, B)** Univariate and multivariate Cox regression analysis of age, gender, grade, stage, TMN stage and risk score. **(C)** The AUC for age, gender, grade, stage, TMN stage and risk score. **(D–F)** LncRNA expression increased with grade, tumor-stage and T-stage. ns p > 0.05, *p < 0.05, **p < 0.01, ***p < 0.001.

A total of 221 HCC patients were included in the independent prognosis analysis, patients without age, gender, grade, tumor-stage, T-stage, N-stage or M-stage information were excluded. We used univariate and multivariate Cox regression analyses to assess the independent risk factors. Several clinicopathological factors, such as the patient’s age, gender, grade, stage, and TMN stage, as well as the immune-related six-lncRNA signature based on the risk score, were included. The forest map illustrated that the hazard ratio (HR) of the risk score and 95% CI were 1.511 and 1.349-1.694 with the univariate Cox regression analysis (p = 1.133e-12) (**Figure 3A**), and 1.442 and 1.271-1.382 using the multivariate Cox regression analysis (p = 1.382e-08) (**Figure 3B**). We applied the ROC curve analysis to illustrate the accuracy of the risk score model. The area under the ROC curve (area under curve, AUC) was measured. The AUC of the risk score and the patient’s age, gender, grade, tumor-stage, T-stage, N-stage, and M-stage are 0.775, 0.454, 0.506, 0.475, 0.743, 0.752, 0.508 and 0.508, respectively (**Figure 3C**). These data suggest that the six-lncRNA signature was an independent prognostic factor in patients suffering from HCC.

### Relationships Between Immune-Related lncRNAs and Clinical Parameters

**Figure 4:**
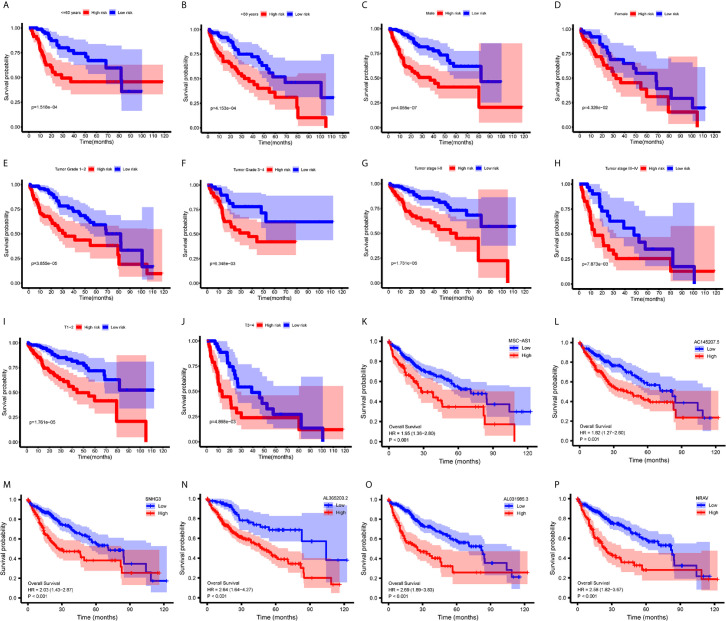
Survival analysis. **(A–J)** Kaplan-Meier survival curves showed that the immune−related lncRNAs signature could predict survival in each stratum of age, gender, stage and T-stage. **(K–P)** Kaplan-Meier survival curves showed that the high lncRNA expressions correlated with worse survival.

To investigate the relationship between immune-related lncRNAs and clinical parameters, we analyzed the correlation of immune-related lncRNAs and the clinical characteristics, such as the patient’s grade (n=337), tumor-stage (n=321), and T-stage (n=340), patients with incomplete clinical information were excluded. We found that lncRNAs increased with grade, tumor-stage, and T-stage (**Figures 3D–F**). Besides, the Kaplan-Meier survival curves showed that the lncRNAs were correlated with worse survival rates in HCC patients (**Figures 4K–P**).

### Stratification Analysis

We performed a stratification analysis according to the clinical parameters of HCC, such as the patient’s age, gender, grade, tumor-stage, and T-stage. Patients with incomplete clinical information were excluded. The Kaplan-Meier survival curves showed that the immune-related lncRNAs signature was correlated with worse survival rates in younger (<= 60 years, n=165, p = 1.518e-04) (**Figure 4A**) or older (>60 years, n=178, p = 4.153e-04) (**Figure 4B**); male (n=233, p= 4.055e-07) (**Figure 4C**) or female (n=110, p = 4.326e-02) (**Figure 4D**); lower grade (n=214, grade 1 - 2, p = 3.855e-05) (**Figure 4E**) or higher grade (n=124, grade 3 – 4, p = 6.348e-03) (**Figure 4F**); stage 1-2 (n=238, p=1.731e−05) (**Figure 4G**) or stage 3-4 (n=83, p = 7.873e-03) (**Figure 4H**); and T1 - 2 (n=252, p = 1.761e-05) (**Figure 4I**) or T3 - 4 (n=89, p = 4.868e-03) (**Figure 4J**) patients. These results suggest that our immune-related lncRNAs signature, based on the risk score, remains a powerful tool for predicting HCC survival in each stratum of age, gender, stage, and T-stage.

### PCA Analysis

**Figure 5:**
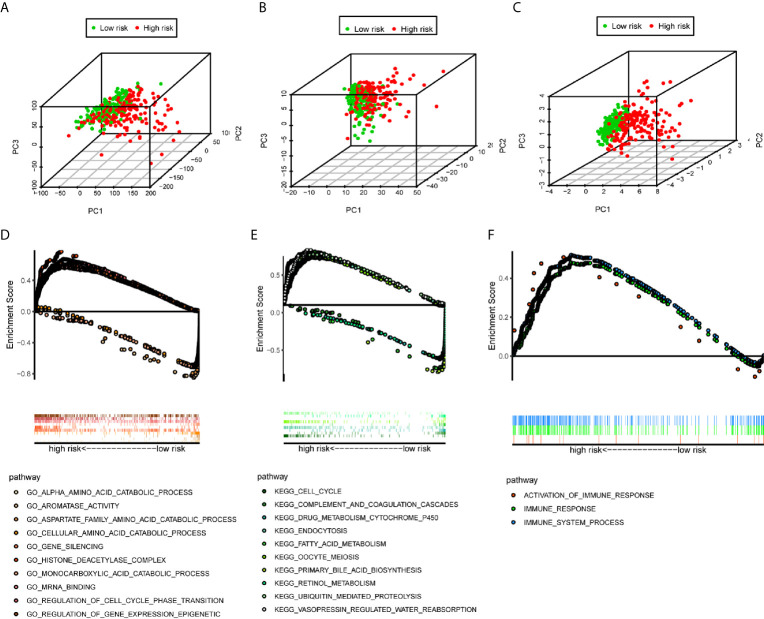
PCA analysis and GSEA analysis. **(A–C)** The results demonstrated that in the six immune-related lncRNAs set, the low-risk group and the high-risk group were separated into two parts better than based on all genes set, all immune-related lncRNAs set. **(D)** GO analysis and **(E)** KEGG analysis results. **(F)** Immune-related responses such as activation of immune response, immune response and immune system process were enriched in high-risk groups.

We used the PCA analysis to investigate the different distribution patterns between low-risk (n=172)and high-risk (n=171) groups, based on different expression profiles. The low-risk and high-risk groups are represented by green and red dots, respectively. **Figures 5A–C** show the PCA results based on all genes set, all immune-related lncRNAs set, and the immune-related six-lncRNA set, respectively. The results demonstrated that in the immune-related six-lncRNA set, the low-risk and high-risk groups were separated into two parts, and the immune status of the patients in the low-risk group was distinguished from those in the high-risk group.

### GSEA Analysis of the Immune-Related lncRNAs Signature

The GSEA indicated that the primarily increased functions of the GO analysis (**Figure 5D**) were the regulation of gene expression epigenetic, gene silencing, histone deacetylase complex, mRNA binding, and regulation of cell cycle phase transition; while the primarily decreased functions of the GO analysis were the aromatase activity, alpha amino acid catabolic process, monocarboxylic acid catabolic process, cellular amino acid catabolic process, and aspartate family amino acid catabolic process. The primarily increased functions of the KEGG analysis (**Figure 5E**) were endocytosis, vasopressin regulated water reabsorption, oocyte meiosis, ubiquitin mediated proteolysis, and cell cycle; while the primarily decreased functions of the KEGG analysis were the retinol metabolism, drug metabolism cytochrome P450, fatty acid metabolism, primary bile acid biosynthesis, and complement and coagulation cascades. Besides, the results showed that the immune-related responses, such as the activation of the immune response, immune response, and immune system process were enriched in the high-risk group compared to the low-risk group (**Figure 5F**).

### Correlation Between the Immune-Related lncRNA and Immune Cell Infiltration

**Figure 6:**
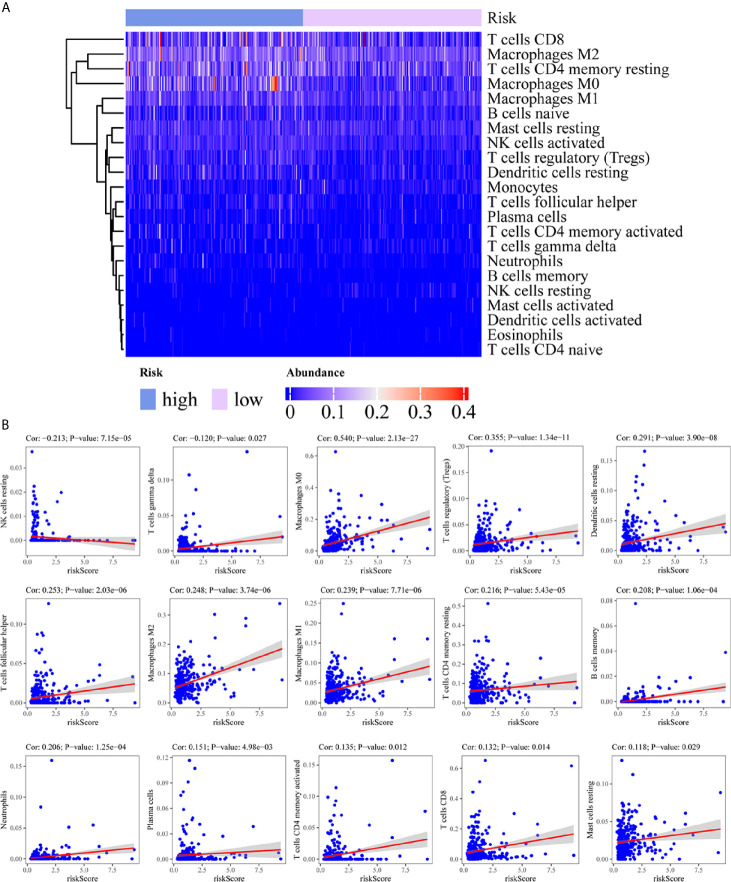
Correlation between the immune-related lncRNA signature and 22 immune cell infiltration using CIBERSORT. **(A)** The heatmap showed the 22 TIICs abundance in high-risk groups and low-risk groups. **(B)** Correlation between immune cell infiltration and this immune-related lncRNA signature.

**Figure 7:**
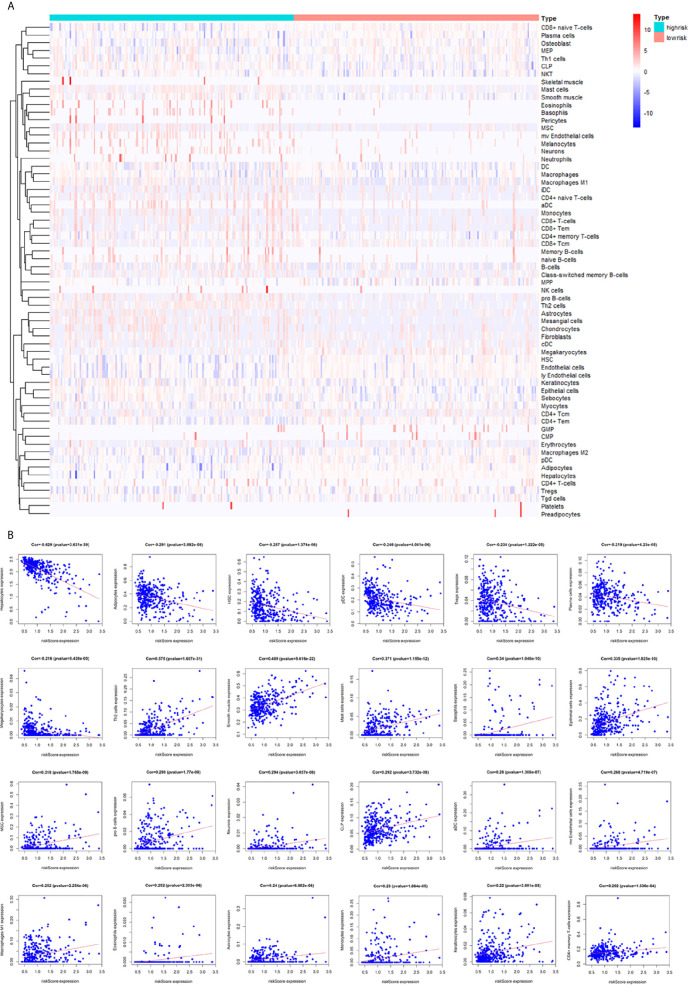
Correlation between the immune-related lncRNA signature and 64 cell heterogeneity using xCell. **(A)** The heatmap showed the 64 cells abundance in high-risk groups and low-risk groups. **(B)** Correlation between cell heterogeneity and this immune-related lncRNA signature.

**Figure 8:**
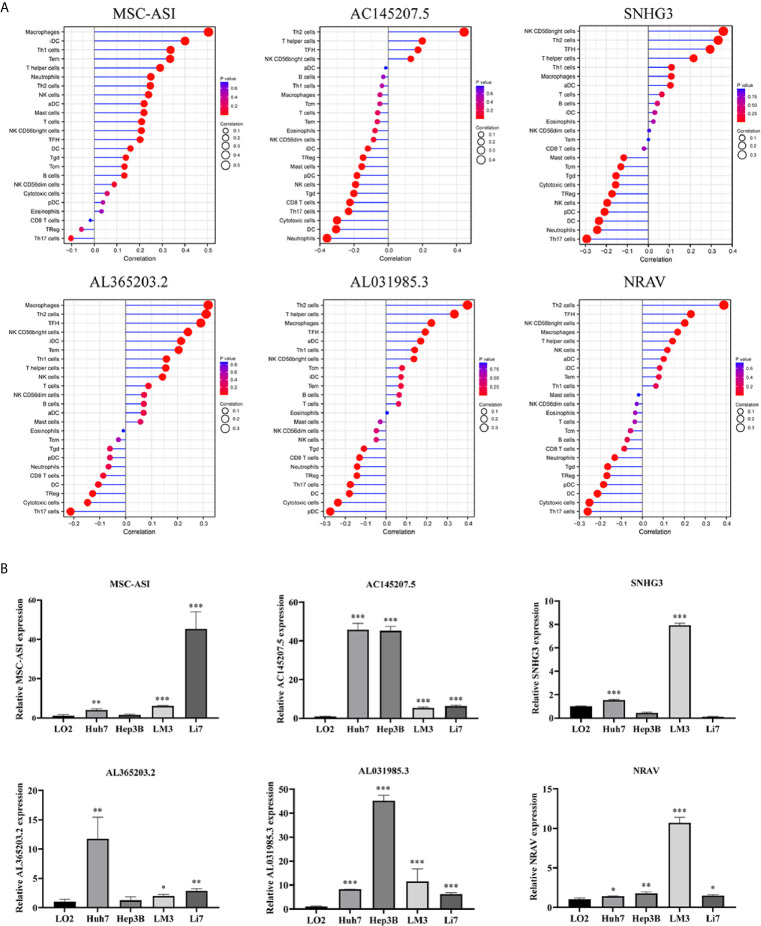
ssGSEA analysis of six immune-related lncRNAs and Expression level of six immune-related lncRNAs in cell lines. **(A)** Correlation between 24 immune cells and six immune-related lncRNAs. **(B)** The expression of the six immune-related lncRNAs in four HCC cell line and one normal human hepatic cell line. *p < 0.05, **p < 0.01, ***p < 0.001.

To assess the relationship between the immune cell infiltration and our six-lncRNA signature, we profiled 22 TIICs, using the CIBERSORT algorithm. The heatmap showed the TIICs abundance in the high-risk (n=171)and low-risk (n=172) groups (**Figure 6A**). Given that these six lncRNAs were related to tumor immunity, we analyzed the correlation between the six-lncRNA signature and the infiltration of immune cell subtypes (**Figure 6B**). The results showed that the most significant positive correlation with the immune infiltration were Macrophages M0, T cells regulatory, Dendritic cells resting, T cells follicular helper, Macrophages M2, Macrophages M1, T cells CD4 memory resting, Neutrophils, Plasma cells, T cells CD4 memory activated, T cells CD8 and Mast cells resting., while the most significant negative correlation with the immune infiltration were NK cells resting and T cells gamma delta. Besides, xCell algorithm showed 64 cells in the high-risk and low-risk group were different (**Figure 7A**). The most significant positive correlation with the risk score were Th2 cells, Smooth muscle, Mast cells, Basophils, Epithelial cells, MSC, pro B-cells, Neurons, CLP, aDC, mv Endothelial cells, Macrophages M1, Eosinophils, Astrocytes, Monocytes, Keratinocytes and CD4+ memory T-cells. The most significant negative correlation with the risk score were Hepatocytes, Adipocytes, HSC, pDC, Tregs, Plasma cells and Megakaryocytes (**Figure 7B**). From ssGSEA we could find that all the lncRNAs were positively correlated with Th2, TFH which could promote cancer progression. While all the lncRNAs were negative correlated with CD8 T cells, Treg and Th 17 cells which could inhibit cancer progression (**Figure 8A**). These findings suggest that the six immune-related lncRNAs were associated with the immune cell infiltration in HCC.

### Expression Level of Six Immune-Related lncRNAs in Cell Lines

We detected the expression levels of six lncRNAs in four human HCC cell lines (Huh7, Hep3B, LM3, Li7) and one normal human hepatic cell line (LO2). The results showed that the six lncRNAs were highly expressed in four HCC cell lines than in normal human hepatic cell line (**Figure 8B**).

### Impact of the Immune-Related lncRNA Signature and Immune Checkpoint Gene Expression on the Clinical Outcomes

**Figure 9:**
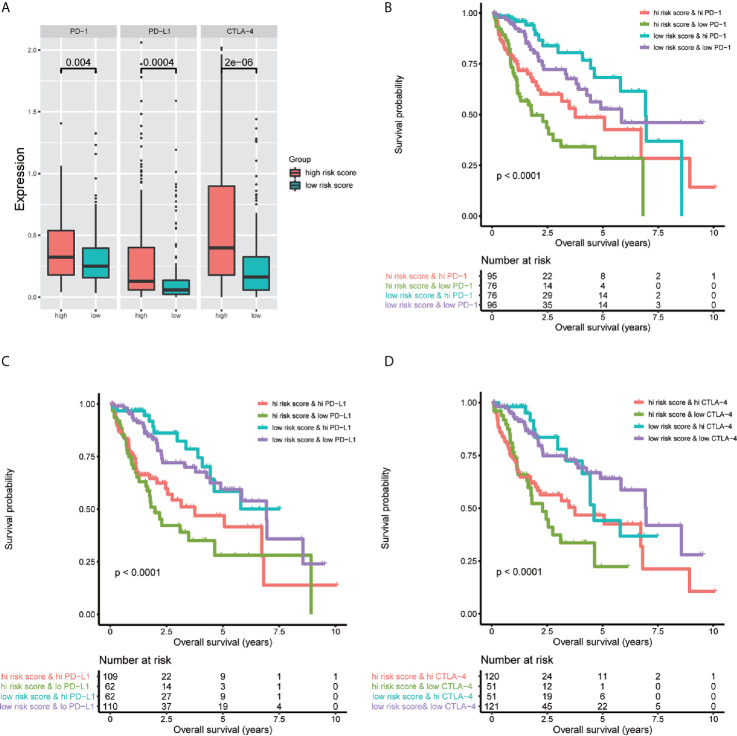
Impact of the immune-related lncRNA signature and immune checkpoint gene expression on clinical outcome. **(A)** relationship between the immune-related lncRNA signature and immune checkpoint genes, the results showed that patients in high-risk group have higher expression of immune checkpoint genes than patients in low-risk group. Kaplan-Meier survival curves of overall survival among four patient groups stratified by the immune-related lncRNA signature and PD-1 **(B)**, PD- L1 **(C)** and CTLA-4 **(D)**.

To investigate the relationship between the immune-related lncRNA signature and immune checkpoint genes, we compared the expression of immune checkpoint genes, including PD1, PD-L1, and CTLA-4, between the high-risk (n=171)and low-risk (n=172) groups. The results showed that patients in the high-risk group have a higher expression of immune checkpoint genes than patients in the low-risk group (**Figure 9A**). Besides, we compared the survival distribution of four patient groups stratified by the high/low-risk score and the high/low immune checkpoint gene expression. Kaplan-Meier survival curves showed that patients in the low-risk group with high PD1 have better survival rates than patients in the high-risk group with high PD1, and patients in the low-risk group with low PD1 present better survival rates than patients in the high-risk group with low PD1 (**Figure 9B**). We observed similar results in the PD-L1 and CTLA-4 stratifying groups (**Figures 9C, D**). These results indicate that the immune-related lncRNA signature may be a potential predictive biomarker of the treatment response to immunotherapy.

## Discussion

Hepatocellular carcinoma is one of the world’s most common malignancies, and although scientists have been investing considerable time, effort, and expenses in research on this cancer, its incidence and mortality keep increasing each year (13). Therefore, it is of great clinical significance to explore the early diagnoses of HCC, accurately predict patients’ prognoses, and determine the potential therapeutic targets and prognostic indicators of HCC.

It is widely known that the TME contains tumor cells, as well as surrounding immune cells, endothelial cells, fibroblasts, extracellular matrix, secreted cytokines, and chemokines, to name a few. Tumors can create a series of favorable conditions for themselves through the TME, and even escape the immune cycle. A recent study has found that cellular immune responses are involved in the development of HCC and may be among the factors influencing a poor prognosis in HCC (14). In this study, we analyzed expression data extracted from the TCGA database using the ESTIMATE method and exposed significant differences in EstimateScore, ImmuneScore, StromalScore, and TumorPurity between the immunity high group and immunity low group of HCC. Besides, we found that the proportions of TIICs varied between the two groups, based on the CIBERSORT method.

With the development of next-generation sequencing technology, numerous genomic and transcriptomic sequences are available in public databases nowadays. Through mining the public database, researchers found that lncRNAs acted as key regulators in the development of cancer in various cellular functions, including proliferation, cell differentiation, and DNA stability, to name a few (15). Although the roles of many lncRNAs in HCC remain elusive, a small part has been extensively investigated. For instance, the HBx-LINE1 activates the Wnt signaling, promotes the HCC development and progression, and correlates with shorter patient survival (16). Meanwhile, lncRNA HULC is upregulated in HCC and promotes HCC growth, metastasis, and drug resistance (17). LncRNA-WRAP53 is an independent prognostic marker in relapse-free survival and may serve as a serum biomarker for the diagnosis and prognosis of HCC (18). These observations point to the considerable potential of lncRNAs as a source of novel targetable molecules for HCC precision therapy and for discovering new diagnostic biomarkers.

Evidence has recently indicated that lncRNAs can regulate immune cell differentiation and function, such as dendritic cell activity, T cell ratio, and metabolism (6), and thus, are potential targets for cancer therapeutics and possess predictive value for survival prognosis (4). Through mining the transcriptome sequencing data *via* the bioinformatics analysis, many studies have already established the lncRNA signature for predicting the prognosis of cancers, including thyroid cancer (19), breast cancer (20), renal cell carcinoma (21), bladder cancer (22), gastric cancer (23), as well as HCC (24). In this study, we aimed to construct an immune-related lncRNAs signature in HCC. We obtained a total of 331 immune-related genes from the Molecular Signatures Database (Immune response M19817, immune system process M13664) and identified 236 immune-related lncRNAs through the correlation analysis. Using the univariate cox regression, we identified six immune-related lncRNAs — MSC-AS1, AC145207.5, SNHG3, AL365203.2, AL031985.3, NRAV — as a prognostic signature for HCC. Using the risk score method, we developed an immune-related six-lncRNA signature, which permitted us to classify HCC patients into a high-risk group and a low-risk group, with significantly different overall survival rates. We analyzed the relationship between a patient’s age, gender, grade, tumor stage, and risk score using the univariate and multivariate Cox regression analyses. The results showed that only the risk score had p < 0.05 in both the univariate and multivariate Cox regression analyses. The AUC of the risk score, at 0.775, was greater than other factors. Such data suggest that the risk score may be an independent prognostic factor in HCC patients. Then, we analyzed the correlation between immune-related lncRNAs and clinical characteristics and found that these lncRNAs increased with grade, tumor-stage, and T-stage. However, we also noticed a decrease in stage IV compared to stage II and III in some lncRNAs. We may attribute this decrease to a few reasons. First, there only were 5 patients suffering from stage IV HCC, which may lead to an obvious deviation. Second, the decrease might be owning to the lncRNA itself, or the patients in stage IV have some particular genetic characteristics. Therefore, further research with expanded patients is needed to verify these results. To investigate the applicability of the signature in different clinical conditions, we performed stratification analyses. Through them, we observed that the signature could assess the risk score in subgroups of HCC patients and predict HCC patients’ survival in each stratum of age, gender, stage, and T-stage. Besides, we employed the GSEA to verify the functional annotation and found that the activation of the immune-related responses, immune response, and immune system process were enriched in high-risk groups.

The immune cell infiltration in the TME may affect tumor cell survival, metastasis, and therapy resistance (25, 26). Using the CIBERSORT method, we conducted a comprehensive analysis of the TME immune cells infiltration landscape through the estimation of the abundance of 22 TIICs in HCC. We found that eosinophils and T cells follicular helper were positively correlated with the lncRNA prognostic signature, while monocytes, NK cells activated, plasma cells, and T cells CD4 memory resting were negatively correlated with the lncRNA prognostic signature. These findings suggest that the immune-related six-lncRNA signature may play a role in the immune infiltration of HCC. Among six immune-related lncRNAs in the signature, several lncRNAs have been reported to be associated with cancer development and prognosis. For example, MSC-AS1 activates Wnt/β-catenin signaling pathway to modulate cell proliferation and migration in kidney renal clear cell carcinoma *via* miR-3924/WNT5A (27). SNHG3 promotes migration, invasion, and epithelial-mesenchymal transition of breast cancer cells through the miR-186-5p/ZEB1 axis (28). However, the underlying mechanism of this association in HCC requires further investigation.

In conclusion, using bioinformatics methods and qRT-PCR experiment, we identified six immune-related lncRNAs that were correlated with HCC progression and prognosis and may be applied as independent prognostic indicators in predicting HCC patients’ survival. Besides, the six immune-related lncRNAs were related to the level of infiltration of tumor-infiltrating immune cells. Therefore, the identification of immune-related lncRNAs may provide new targets for the research of the molecular mechanisms and immunotherapy of HCC in the future.

## Data Availability Statement

The original contributions presented in the study are included in the article/**Supplementary Material**. Further inquiries can be directed to the corresponding authors.

## Author Contributions

ZP and ZY conceived and designed the present study. ZP collect and performed the experiments. ZP and WL analyzed and wrote the manuscript. LY check and revised the manuscript. All authors contributed to the article and approved the submitted version.

## Funding

This study was funded by National Natural Science Foundation of China (81872255), Key Medical Personnel Foundation of Jiangsu (2016KJQWZDRC-03), China Postdoctoral Science Foundation, General Project (2017M621801), Nantong Science and Technology Project (MS12019024, MS12018048).

## Conflict of Interest

The authors declare that the research was conducted in the absence of any commercial or financial relationships that could be construed as a potential conflict of interest.
